# Supplementary material for: Molecular anatomy of the pre-primitive-streak chick embryo
Source: Open Biol. 2020 Feb 12;10(2):190299. doi: 10.1098/rsob.190299 (PMC7058932; doi:10.1098/rsob.190299)
Supplement: Supplementary Figures and Tables [file rsob190299supp1.pdf]

**Title:** Molecular anatomy of the pre-primitive-streak chick embryo

**Authors:** Hyung Chul Lee[1], Hui-Chun Lu[1], Mark Turmaine[1], Nidia M. M. Oliveira[1], Youwen Yang[1,2], Irene De Almeida[1] and Claudio D. Stern[1,*]

[1] Department of Cell and Developmental Biology, University College London, Gower Street, London WC1E 6BT, UK

* c.stern@ucl.ac.uk

**Journal name:** Open Biology

**Article DOI:** 10.11098/rsob190299

**Supplementary figures**

**Figure S1. Progression of hypoblast in the prestreak stage embryos.** Sagittal sections on anteroposterior axis stained with PKCζ and DAPI shows gradual progression and length of hypoblast sheet.

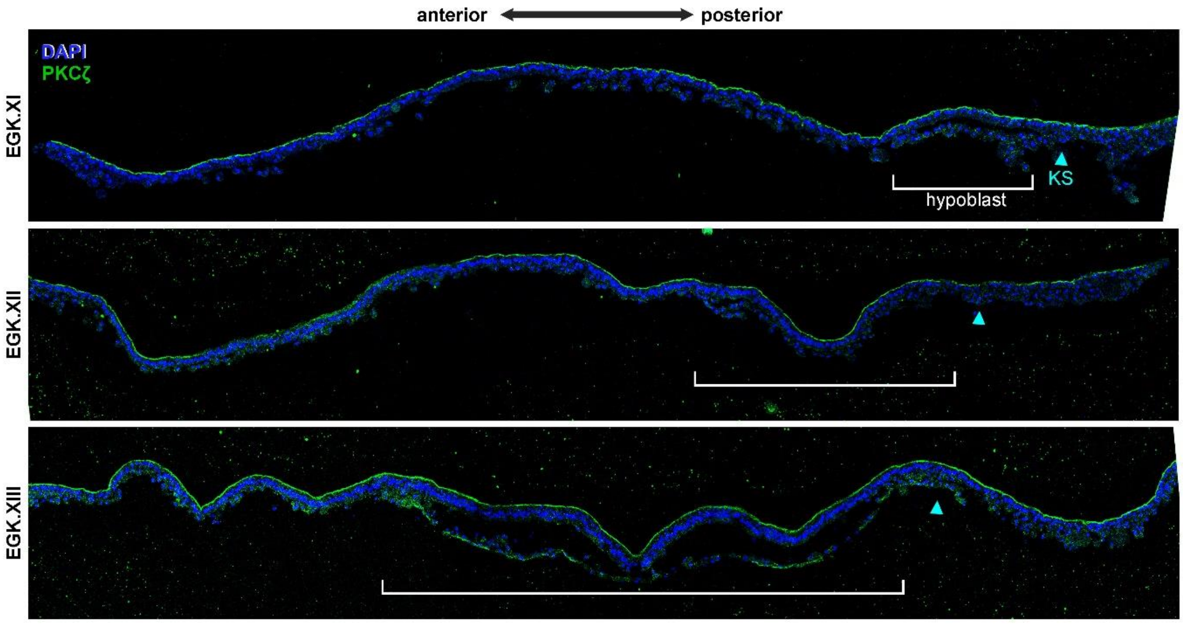

**Figure S2. PAR3 and PKCζ don't show regional difference in expression.** (A) No difference in expression was seen for both PAR3 and PKCζ between anterior and posterior sides in sections of EGK XI-XIII. (B) There is also no difference between the AP and the AO. Scale bars, 100 μm.

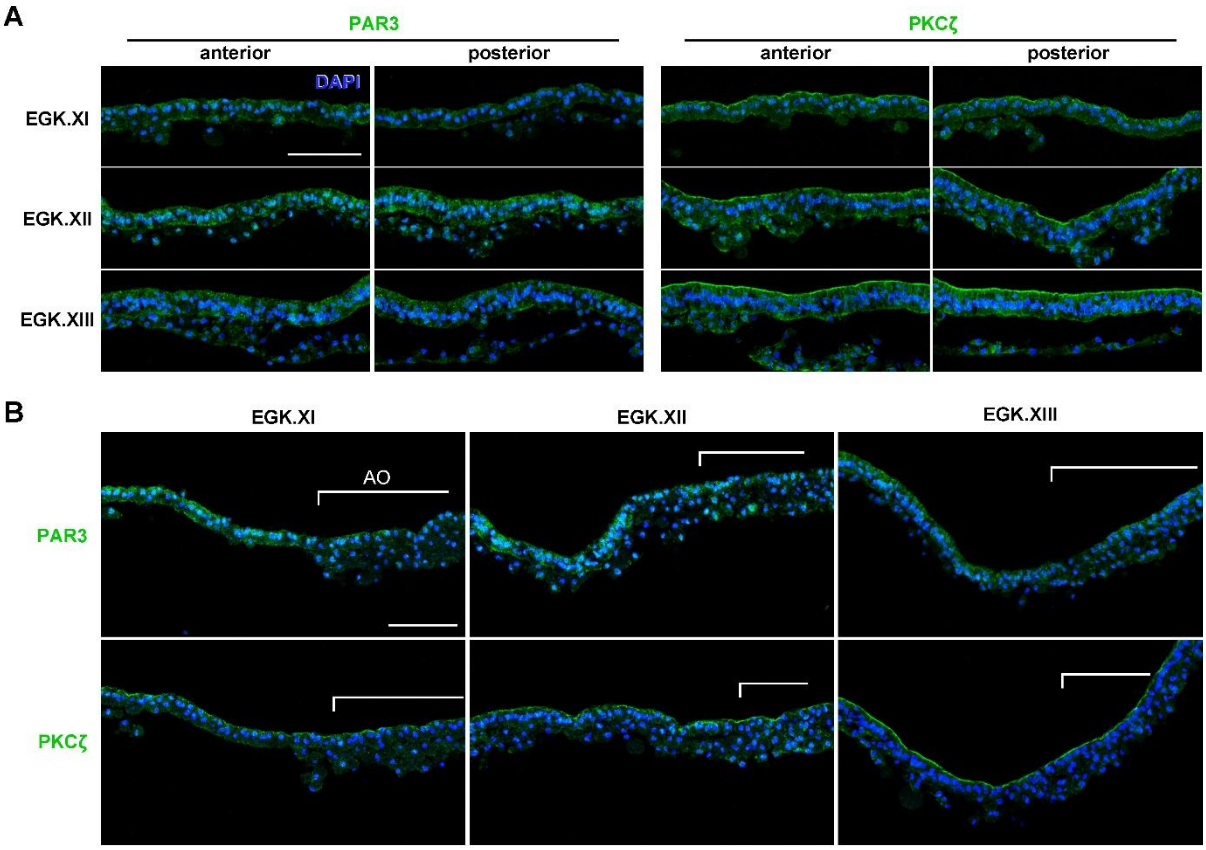

**Figure S3. RNAseq results of different regions of prestreak chick embryos at EGK.XII-III.** (A) A total of 12 samples was collected for RNAseq (table). The precise location for sampling is shown with dorsal and sagittal section views. Arrows on the right side denote orientation of an embryo: A, anterior; P, posterior; L, left; R, right; D, dorsal; V, ventral. area opaca, AO; marginal zone, MZ; area pellucida, AP; Koller's sickle, KS; germ wall, GW. (B) Hierarchical clustering heat map of RNA-seq data. Color bar indicates the level of gene expression: red, high expression; blue, low expression. area opaca, AO; marginal zone, MZ; area pellucida, AP; Koller's sickle, KS; germ wall, GW. Lowercase a and p indicate anterior and posterior, respectively (C) Principal components analysis of RNA-seq. Colored circles indicate distinguished embryonic regions: red, AO; yellow, MZ; green, AP; blue, lower layers.

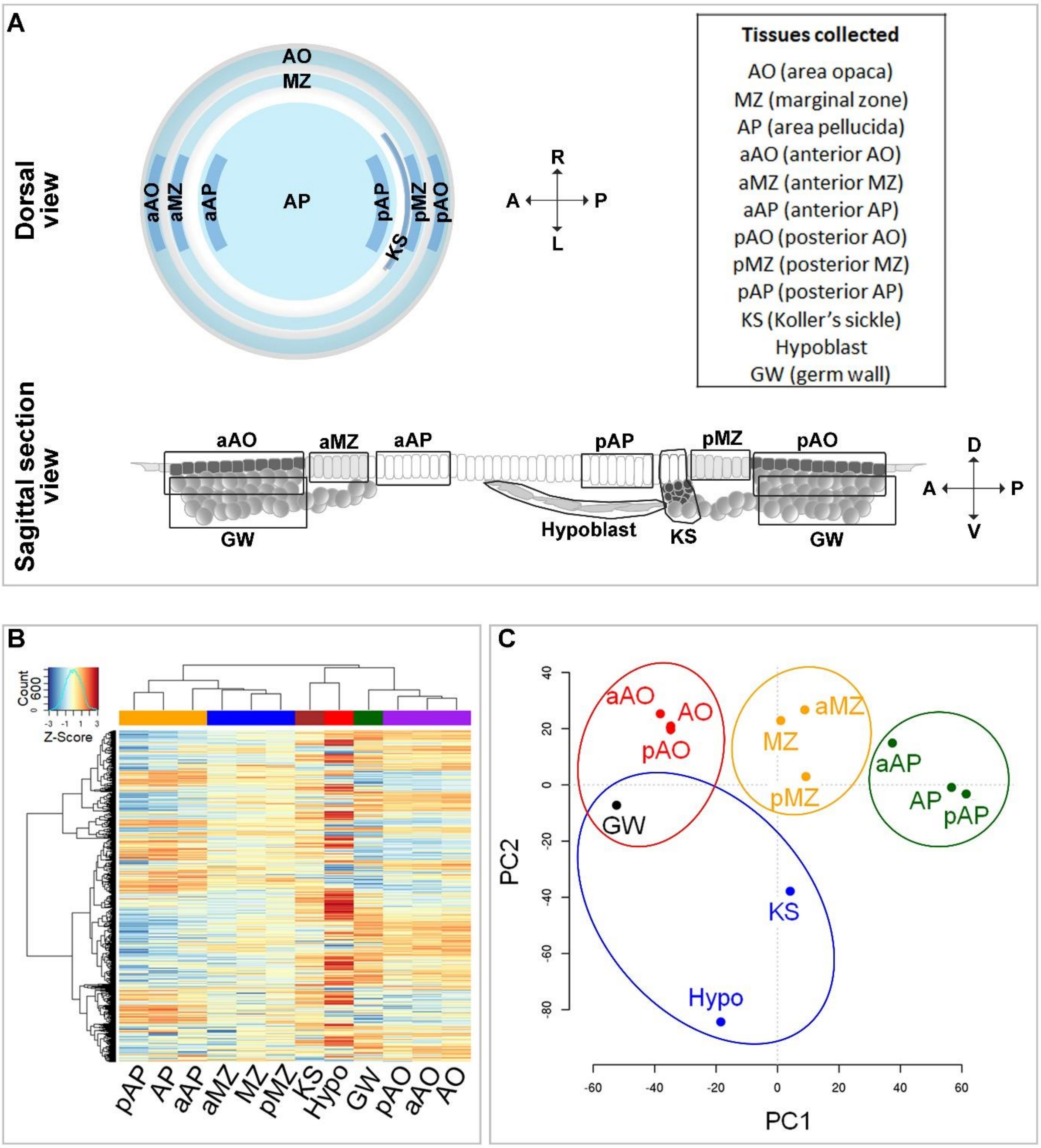

**Figure S4. Expression of the other listed genes in Supplementary table S3-8, which are not suitable for a marker due to low expression.** Expression pattern of genes revealed by in situ hybridization in whole mount at three stages: EGK X-XI, EGK XII-XIII, EGK XIV-HH 2. Whole embryos shown anterior side-up. Note that *CDC42EP2* and *VLDLR* have undetectable level of expression, while *MIXL1* is not available for its RNA-probe.

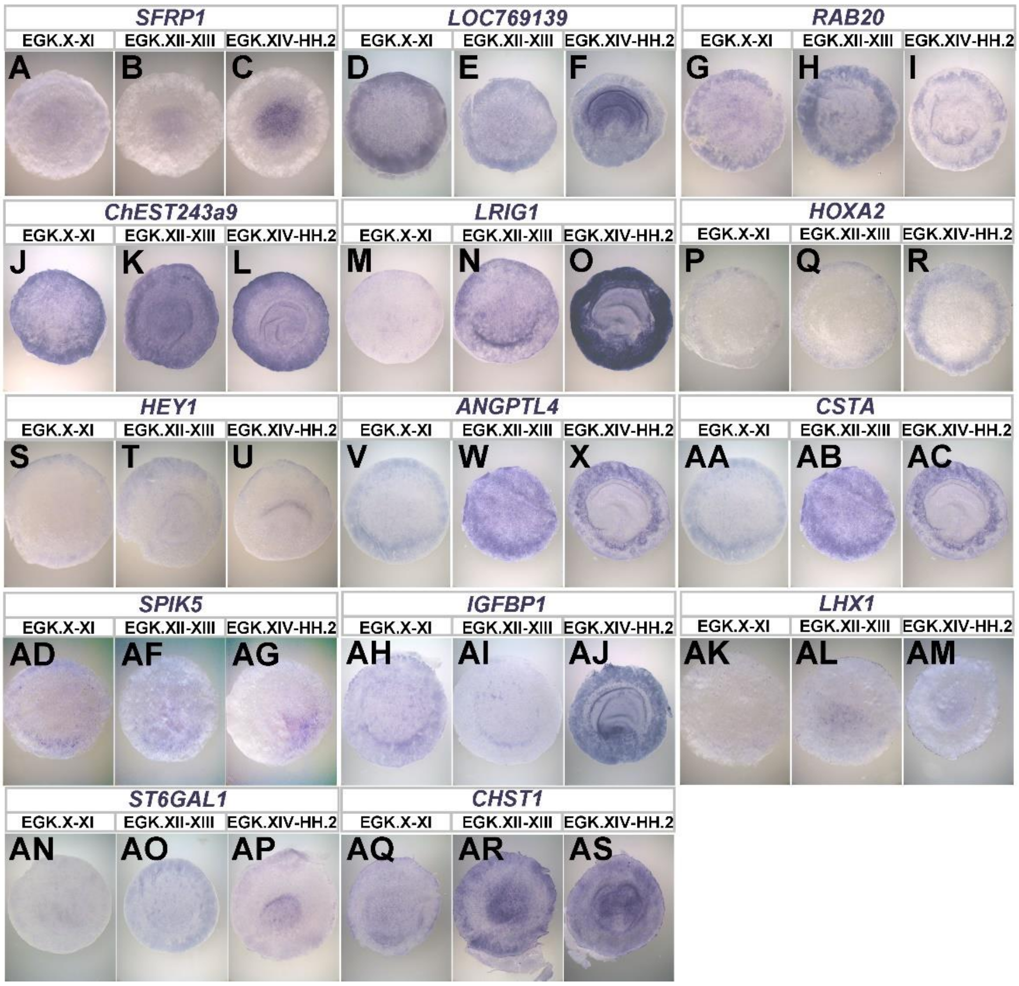

**Figure S5. Differential cell polarization of the pMZ compared to the pAP.** Double staining of ASTL mRNA (A) for labeling the MZ and RAC1 protein (B-D) for showing cell shape reveals distinct cell polarization in the pMZ. The boxed regions in (A) and (B) (the upper for the pAP and the lower for the pMZ) are enlarged in (C) and (D), respectively. White arrows indicate the KS. Scale bar, 100 μm

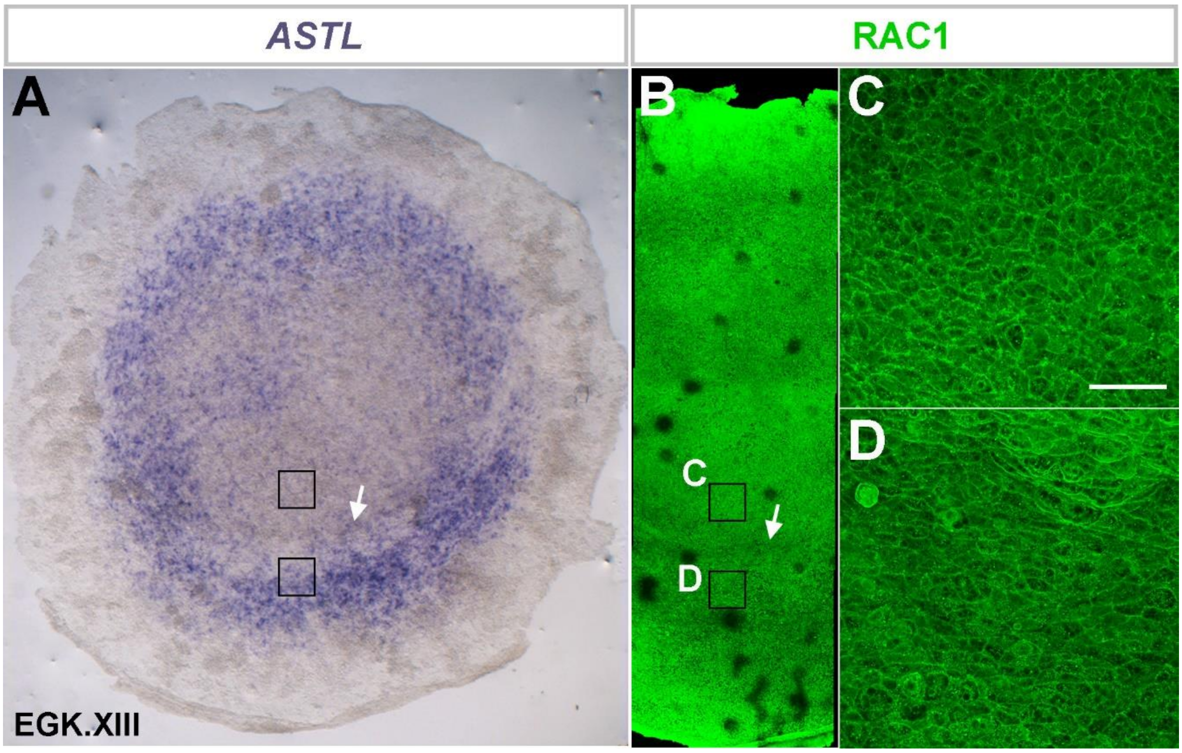

**Figure S6. Expression pattern of *NODAL* and *cVG1* in the prestreak stage embryos.** mRNA expression patterns of *NODAL* (A) and *cVG1* (B) are shown by wholemount in situ hybridization and by paraffin sectioned images (A', A'', B', and B'') at three different stages; EGK.X-XI, EGK.XII-XIII, EGK.XIV-HH.2. Embryonic regions with distinct expression are denoted on the panels of EGK.XII-XIII. Dotted lines indicate the position of sectioning. Orientation: embryos, anterior side-up; sections, posterior side-right. Scale bars, 100 µm.

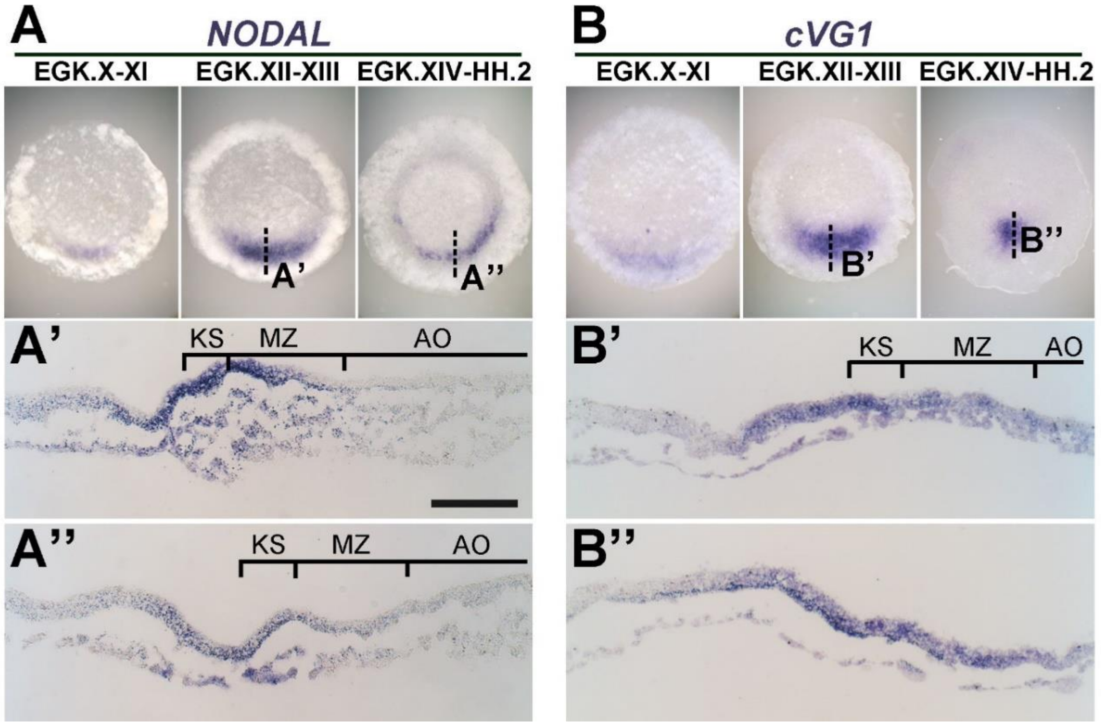

Supplementary table 1. Sampling details for RNAseq

| ID samples | Description | No. of embryos | ng/µL | RIN | Elution volume |
|---|---|---|---|---|---|
| **AO** | area opaca | 12 | 104 | 7.4 | 30 |
| **MZ** | marginal zone | 16 | 111 | 7.5 | 30 |
| **AP** | area pellucida | 12 | 226 | 6.7 | 30 |
| **aAO** | anterior AO | 20 | 33.6 | 7.1 | 30 |
| **aMZ** | anterior MZ | 20 | 18.5 | 7 | 30 |
| **aAP** | anterior AP | 20 | 29.2 | 8.1 | 30 |
| **pAO** | posterior AO | 20 | 30.1 | 7.5 | 30 |
| **pMZ** | posterior MZ | 20 | 23.2 | 7.1 | 30 |
| **pAP** | posterior AP | 20 | 50.5 | 5.8 | 30 |
| **KS** | Koller's sickle | 20 | 40.4 | 6.1 | 30 |
| **Hypoblast** | hypoblast | 20 | 16.4 | 7.7 | 30 |
| **GW** | germ wall | 20 | 13.1 | 8.2 | 30 |

Supplementary table 2. Number of cells (embryos) used in quantification of average aspect ratio in Fig. 2

**For Fig. 2 D-E**

| | X-XI | XII | XIII |
|---|---|---|---|
| **AP** | 2119 (13) | 614 (3) | 768 (3) |
| **AO** | 99 (6) | 32 (2) | 68 (2) |

**For Fig. 2 J-K**

| | ant | pos |
|---|---|---|
| **X** | 983 (5) | 863 (5) |
| **XIII** | 533 (3) | 616 (2) |

Supplementary table 3. List of MZ enriched genes

| | FPKM | | | Fold change* | | | | | |
|---|---|---|---|---|---|---|---|---|---|
| **Gene name** | **MZ** | **aMZ** | **pMZ** | **MZ/AO** | **MZ/AP** | **aMZ/aAO** | **aMZ/aAP*** | **pMZ/pAO** | **pMZ/pAP** |
| ASTL | 293.324 | 152.801 | 292.207 | 4.683 | 2.105 | 3.780 | 0.498 | 2.394 | 1.584 |
| ARL4C | 212.265 | 167.121 | 302.080 | 1.640 | 1.485 | 1.665 | 1.397 | 2.236 | 1.627 |
| TBX6 | 90.271 | 24.811 | 189.033 | 3.302 | 1.649 | 2.375 | 0.865 | 3.571 | 2.361 |
| CDC42EP4 | 38.113 | 27.147 | 38.554 | 2.129 | 2.197 | 1.612 | 1.527 | 1.472 | 1.446 |
| MIXL1 | 27.252 | 20.281 | 114.762 | 6.697 | 1.437 | 8.123 | 4.802 | 14.292 | 2.839 |

*Threshold; Fold change > 1.4 except comparison between aMZ and aAP

Supplementary table 4. List of AP enriched genes

| Gene name | FPKM | Fold change* | | | | | |
|---|---|---|---|---|---|---|---|
| | AP | AP/AO | AP/MZ | aAP/aAO | aAP/aMZ | pAP/pAO | pAP/pMZ |
| RNH1 | 306.396 | 6.534 | 3.358 | 3.933 | 1.938 | 7.387 | 3.406 |
| MAFA | 104.023 | 7.117 | 4.205 | 4.463 | 1.604 | 9.303 | 4.769 |
| ADMP | 82.4662 | 302.806 | 8.135 | 22.937 | 1.936 | 203.671 | 5.987 |
| SFRP1 | 70.1655 | 21.325 | 5.798 | 20.552 | 4.867 | 19.080 | 4.826 |
| NKAIN4 | 56.2542 | 11.457 | 4.537 | 11.428 | 2.364 | 12.894 | 3.262 |
| LOC769139 (feather keratin 1-like) | 52.6645 | 10.455 | 4.004 | 7.405 | 2.261 | 16.797 | 8.498 |
| LFNG | 46.5435 | 13.247 | 4.076 | 11.380 | 2.892 | 11.633 | 3.867 |

* Threshold; Fold change > 3 except comparison between aAP and aMZ

Supplementary table 5. List of AO enriched genes

| Gene name | FPKM | | | | Fold change* | | | | | |
|---|---|---|---|---|---|---|---|---|---|---|
| | AO | aAO | pAO | GW | AO/MZ | AO/AP | aAO/aMZ | aAO/aAP | pAO/pMZ | pAO/pAP |
| DLL1 | 102.039 | 110.579 | 92.892 | 279.471 | 8.561 | 490.146 | 13.132 | 107.351 | 11.827 | 792.596 |
| RAB20 | 71.599 | 77.869 | 80.821 | 161.129 | 3.382 | 3.956 | 3.141 | 4.393 | 3.175 | 3.820 |
| ENSGALG00000042427 (CR524387/ChEST243a9) | 55.052 | 66.915 | 60.638 | 74.3593 | 3.981 | 3.045 | 3.489 | 4.559 | 3.661 | 3.065 |
| LRIG1 | 51.465 | 42.642 | 42.214 | 86.6624 | 4.774 | 18.635 | 5.799 | 6.537 | 3.538 | 30.218 |
| DOC2B | 34.220 | 38.193 | 34.828 | 90.0425 | 3.825 | 8.116 | 5.209 | 7.594 | 4.031 | 10.236 |
| VLDLR | 32.377 | 41.066 | 39.651 | 52.602 | 3.303 | 7.781 | 3.622 | 5.756 | 7.758 | 9.667 |
| HOXA2 | 31.400 | 28.994 | 22.881 | 40.834 | 4.177 | 53.797 | 4.757 | 29.407 | 4.614 | 74.277 |

* Threshold; Fold change > 3

Supplementary table 6. List of GW enriched genes

| Gene name | FPKM | Fold change* | | | | | | | | | | |
|---|---|---|---|---|---|---|---|---|---|---|---|---|
| | **GW** | **GW/AO** | **GW/MZ** | **GW/AP** | **GW/aAO** | **GW/aMZ** | **GW/aAP** | **GW/pAO** | **GW/pMZ** | **GW/pAP** | **GW/KS** | **GW/Hypoblast** |
| DKK1 | 804.421 | 2.775 | 10.525 | 62.838 | 2.573 | 23.665 | 53.478 | 2.685 | 4.854 | 34.622 | 2.510 | 2.387 |
| DLL1 | 279.471 | 2.739 | 23.448 | 1342.442 | 2.527 | 33.188 | 271.313 | 3.009 | 35.583 | 2384.565 | 48.639 | 71.977 |
| WNT8A (cWNT8C) | 200.451 | 2.695 | 10.677 | 385.635 | 2.546 | 17.044 | 265.939 | 3.168 | 6.086 | 258.778 | 4.356 | 38.646 |
| DOC2B | 90.043 | 2.631 | 10.064 | 21.354 | 2.358 | 12.281 | 17.903 | 2.585 | 10.421 | 26.464 | 11.954 | 5.695 |
| HEY1 | 87.293 | 2.399 | 5.789 | 14.361 | 2.262 | 6.183 | 10.297 | 2.397 | 6.358 | 12.043 | 6.084 | 4.502 |
| ANGPTL4 | 50.699 | 2.359 | 3.479 | 2.314 | 3.138 | 5.444 | 4.212 | 2.502 | 5.139 | 3.287 | 3.075 | 3.322 |

* Threshold; Fold change > 2

Supplementary table 7. List of KS enriched genes

| Gene name | FPKM | Fold change* | | | | | | | | | |
|---|---|---|---|---|---|---|---|---|---|---|---|
| | KS | KS/AO | KS/MZ | KS/AP | KS/aAO | KS/aMZ | KS/aAP | KS/pAO | KS/pMZ | KS/pAP | KS/Hypoblasts |
| PITX2 | 143.614 | 7.914 | 5.858 | 11.839 | 13.224 | 13.439 | 14.022 | 2.376 | 2.242 | 4.043 | 2.084 |
| CHRD | 117.763 | 127.637 | 13.780 | 7.772 | 266.686 | 10.355 | 50.639 | 68.254 | 2.507 | 2.796 | 2.566 |
| CSTA | 48.238 | 4.208 | 9.835 | 9.067 | 4.817 | 13.105 | 20.361 | 3.169 | 4.191 | 6.242 | 2.900 |
| SPIK5 | 30.460 | 13.311 | 27.318 | 153.651 | 24.488 | 62.184 | 231.864 | 4.488 | 3.844 | 109.172 | 13.139 |
| IGFBP1 | 15.121 | 32.652 | 24.379 | 68.799 | 17.978 | 48.726 | 16.368 | 13.760 | 12.525 | 33.547 | 2.594 |

* Threshold; Fold change > 2

Supplementary table 8. List of hypoblast enriched genes

| Gene name | FPKM | Fold change* | | | | | | | | | | |
|---|---|---|---|---|---|---|---|---|---|---|---|---|
| | **Hypoblast** | **Hypoblast /AO** | **Hypoblast /MZ** | **Hypoblast /AP** | **Hypoblast /aAO** | **Hypoblast /aMZ** | **Hypoblast /aAP** | **Hypoblast /pAO** | **Hypoblast /pMZ** | **Hypoblast /pAP** | **Hypoblast /KS** | **Hypoblast /GW** |
| HHEX | 865.737 | 288.950 | 318.273 | 40.845 | 335.791 | 245.972 | 80.792 | 266.262 | 140.450 | 35.771 | 13.753 | 65.590 |
| LOC417741 | 531.92 | 166.950 | 199.559 | 34.358 | 180.023 | 76.222 | 39.268 | 185.403 | 171.954 | 50.532 | 19.688 | 44.644 |
| LHX1 | 150.074 | 468.019 | 283.602 | 46.174 | 967.283 | 283.419 | 101.614 | 920.394 | 70.080 | 49.758 | 10.744 | 70.398 |
| ST6GAL1 | 107.216 | 103.505 | 127.525 | 14.560 | 105.666 | 133.659 | 48.782 | 169.485 | 76.107 | 27.838 | 16.188 | 42.604 |
| CHST1 | 89.599 | 102.995 | 68.779 | 33.584 | 105.263 | 70.757 | 51.911 | 162.761 | 86.868 | 40.455 | 13.282 | 47.425 |

* Threshold; Fold change > 10

Supplementary table 9. List of source of probes for *in situ* hybridisation

| Gene name | Source |
|---|---|
| ADMP | Torlopp et al., 2014, *eLife* |
| ANGPTL4 | ChEST714e17 (Source Bioscience) |
| ARL4C | ChEST901O6 (Source Bioscience) |
| ASTL | ChEST817d16 (Source Bioscience) |
| CDC42EP2 | ChEST553l7 (Source Bioscience) |
| ChEST243a9 | ChEST243a9 (Source Bioscience) |
| CHRD | Streit et al., 1998, *Development* |
| CHST1 | ChEST540g14 (Source Bioscience) |
| CSTA | ChEST821f15 (Source Bioscience) |
| cVG1 | Shah et al., 1997, *Development* |
| DKK1 | Foley et al., 2000, *Development* |
| DLL1 | This study |
| DOC2B | ChEST908m8 (Source Bioscience) |
| HEY1 | Leimeister et al., 2000, *Dev Biol* |
| HHEX | Yatskievych et al., 1999, *Mech Dev* |
| HOXA2 | ChEST671c8 (Source Bioscience) |
| IGFBP1 | ChEST247h2 (Source Bioscience) |
| LFNG | Laufer et al., 1997, *Nature* |
| LHX1 | ChEST389n6 (Source Bioscience) |
| LOC417741 | ChEST714o24 (Source Bioscience) |
| LOC769139 | ChEST675l8 (Source Bioscience) |
| LRIG1 | ChEST252h22 (Source Bioscience) |
| MAFA | Torlopp et al., 2014, *eLife* |
| NKAIN4 | ChEST110n2 (Source Bioscience) |
| NODAL | Levin et al., 1995, *Cell* |
| PITX2 | Zhu et al., 1999, *Curr Biol* |
| RAB20 | ChEST228j14 (Source Bioscience) |
| RNH1 | ChEST73n20 (Source Bioscience) |
| SFRP1 | Esteve et al., 2003, *J Cell Sci* |
| SPIK5 | ChEST157b15 (Source Bioscience) |
| ST6GAL1 | ChEST663h10 (Source Bioscience) |
| TBX6 | Torlopp et al., 2014, *eLife* |
| VLDLR | ChEST263f6 (Source Bioscience) |
| WNT8C | Hume and Dodd, 1993, *Development* |
